# Health benefits from the rapid reduction in ambient exposure to air pollutants after China's clean air actions: progress in efficacy and geographic equality

**DOI:** 10.1093/nsr/nwad263

**Published:** 2023-10-09

**Authors:** Tao Xue, Ruohan Wang, Meng Wang, Yanying Wang, Dan Tong, Xia Meng, Conghong Huang, Siqi Ai, Fangzhou Li, Jingyuan Cao, Mingkun Tong, Xueqiu Ni, Hengyi Liu, Jianyu Deng, Hong Lu, Wei Wan, Jicheng Gong, Shiqiu Zhang, Tong Zhu

**Affiliations:** Institute of Reproductive and Child Health, National Health Commission Key Laboratory of Reproductive Health/Department of Epidemiology and Biostatistics, Ministry of Education Key Laboratory of Epidemiology of Major Diseases (PKU), School of Public Health, Peking University Health Science Centre, Beijing100191, China; State Environmental Protection Key Laboratory of Atmospheric Exposure, and Health Risk Management and Center for Environment and Health, Peking University, Beijing100871, China; Advanced Institute of Information Technology, Peking University, Hangzhou311215, China; Institute of Reproductive and Child Health, National Health Commission Key Laboratory of Reproductive Health/Department of Epidemiology and Biostatistics, Ministry of Education Key Laboratory of Epidemiology of Major Diseases (PKU), School of Public Health, Peking University Health Science Centre, Beijing100191, China; Department of Epidemiology and Environmental Health, School of Public Health and Health Professions, University at Buffalo, Buffalo, NY14214, USA; State Key Joint Laboratory of Environment Simulation and Pollution Control, College of Environmental Sciences and Engineering, Peking University, Beijing100871, China; Department of Earth System Science, Tsinghua University, Beijing100084, China; School of Public Health, Key Laboratory of Public Health Safety of the Ministry of Education, and Key Laboratory of Health Technology Assessment of the Ministry of Health, Fudan University, Shanghai200433, China; College of Land Management, Nanjing Agricultural University, Nanjing 210095, China; National & Local Joint Engineering, Research Center for Rural Land Resources Use and Consolidation, Nanjing 210095, China; State Key Joint Laboratory of Environment Simulation and Pollution Control, College of Environmental Sciences and Engineering, Peking University, Beijing100871, China; State Environmental Protection Key Laboratory of Atmospheric Exposure, and Health Risk Management and Center for Environment and Health, Peking University, Beijing100871, China; State Key Joint Laboratory of Environment Simulation and Pollution Control, College of Environmental Sciences and Engineering, Peking University, Beijing100871, China; State Environmental Protection Key Laboratory of Atmospheric Exposure, and Health Risk Management and Center for Environment and Health, Peking University, Beijing100871, China; State Key Joint Laboratory of Environment Simulation and Pollution Control, College of Environmental Sciences and Engineering, Peking University, Beijing100871, China; State Environmental Protection Key Laboratory of Atmospheric Exposure, and Health Risk Management and Center for Environment and Health, Peking University, Beijing100871, China; Institute of Reproductive and Child Health, National Health Commission Key Laboratory of Reproductive Health/Department of Epidemiology and Biostatistics, Ministry of Education Key Laboratory of Epidemiology of Major Diseases (PKU), School of Public Health, Peking University Health Science Centre, Beijing100191, China; Institute of Reproductive and Child Health, National Health Commission Key Laboratory of Reproductive Health/Department of Epidemiology and Biostatistics, Ministry of Education Key Laboratory of Epidemiology of Major Diseases (PKU), School of Public Health, Peking University Health Science Centre, Beijing100191, China; Institute of Reproductive and Child Health, National Health Commission Key Laboratory of Reproductive Health/Department of Epidemiology and Biostatistics, Ministry of Education Key Laboratory of Epidemiology of Major Diseases (PKU), School of Public Health, Peking University Health Science Centre, Beijing100191, China; Institute of Reproductive and Child Health, National Health Commission Key Laboratory of Reproductive Health/Department of Epidemiology and Biostatistics, Ministry of Education Key Laboratory of Epidemiology of Major Diseases (PKU), School of Public Health, Peking University Health Science Centre, Beijing100191, China; Institute of Reproductive and Child Health, National Health Commission Key Laboratory of Reproductive Health/Department of Epidemiology and Biostatistics, Ministry of Education Key Laboratory of Epidemiology of Major Diseases (PKU), School of Public Health, Peking University Health Science Centre, Beijing100191, China; Clean Air Asia, Beijing100600, China; State Key Joint Laboratory of Environment Simulation and Pollution Control, College of Environmental Sciences and Engineering, Peking University, Beijing100871, China; State Environmental Protection Key Laboratory of Atmospheric Exposure, and Health Risk Management and Center for Environment and Health, Peking University, Beijing100871, China; State Key Joint Laboratory of Environment Simulation and Pollution Control, College of Environmental Sciences and Engineering, Peking University, Beijing100871, China; State Environmental Protection Key Laboratory of Atmospheric Exposure, and Health Risk Management and Center for Environment and Health, Peking University, Beijing100871, China; State Key Joint Laboratory of Environment Simulation and Pollution Control, College of Environmental Sciences and Engineering, Peking University, Beijing100871, China; State Environmental Protection Key Laboratory of Atmospheric Exposure, and Health Risk Management and Center for Environment and Health, Peking University, Beijing100871, China

**Keywords:** clean air action, public health, inequality, fine particulate matter, ozone

## Abstract

Clean air actions (CAAs) in China have been linked to considerable benefits in public health. However, whether the beneficial effects of CAAs are equally distributed geographically is unknown. Using high-resolution maps of the distributions of major air pollutants (fine particulate matter [PM_2.5_] and ozone [O_3_]) and population, we aimed to track spatiotemporal changes in health impacts from, and geographic inequality embedded in, the reduced exposures to PM_2.5_ and O_3_ from 2013 to 2020. We used a method established by the Global Burden of Diseases Study. By analyzing the changes in loss of life expectancy (LLE) attributable to PM_2.5_ and O_3_, we calculated the gain of life expectancy (GLE) to quantify the health benefits of the air-quality improvement. Finally, we assessed the geographic inequality embedded in the GLE using the Gini index (GI). Based on risk assessments of PM_2.5_ and O_3_, during the first stage of CAAs (2013 to 2017), the mean GLE was 1.87 months. Half of the sum of the GLE was disproportionally distributed in about one quarter of the population exposed (GI 0.44). During the second stage of CAAs (2017 to 2020), the mean GLE increased to 3.94 months and geographic inequality decreased (GI 0.18). According to our assessments, CAAs were enhanced, from the first to second stages, in terms of not only preventing premature mortality but also ameliorating health inequalities. The enhancements were related to increased sensitivity to the health effects of air pollution and synergic control of PM_2.5_ and O_3_ levels. Our findings will contribute to optimizing future CAAs.

## Teaser

This study finds that during the first stage of clean air actions in China (2013 to 2017), the mean gain of life expectancy (GLE) was 1.87 months. Half of the sum of the GLE was disproportionally distributed in about one quarter of the population exposed (Gini Index: 0.44). During the second stage (2017 to 2020), the mean GLE increased to 3.94 months and geographic inequality decreased (Gini Index: 0.18). Clean air actions were enhanced, from the first to second stages, in terms of not only preventing premature mortality but also ameliorating health inequalities in China.

## INTRODUCTION

Clean air actions (CAAs) in China from 2013 to 2020 improved air quality [1–3], and may have prevented premature mortality caused by air pollution exposure. In the first stage of CAA implementation (2013 to 2017), the focus was on reducing primary emissions of fine particulate matter (PM_2.5_) in the Beijing-Tianjin-Hebei region (BTH), the Yangtze River Delta (YRD), the Pearl River Delta (PRD) and other regions. The key measures during this stage included controlling industrial emissions, promoting the use of clean energy sources, improving vehicle emissions standards, and optimizing air-quality monitoring systems [4]. During this stage, a nationwide monitoring network has also been established, which provided key inputs for accurate assessments on exposure to air pollutants and their health impacts. The second stage (2018 to 2020) aimed to further improve air quality nationwide. Tailored measures (e.g. reducing emissions of volatile organic compounds [VOCs]) targeting multiple air pollutants, including PM_2.5_ and O_3_, were implemented [5]. Overall, CAAs have markedly reduced air pollution levels in China, thereby significantly enhancing public health. From 2013 to 2020, a 48% decrease in PM_2.5_ concentration was associated with a 21% reduction in attributable deaths (1.75 [95% confidence interval 1.64–1.85] million in 2013 to 1.39 [95% confidence interval 1.27–1.51] million in 2020) [3]. CAAs were associated with improvements in multiple health indicators, including lung function [6], lung-cancer incidence [7] and survival rate [8], kidney function [9], blood lipids [10], physical functions [11], household medical expenditure [12], and mental health [13–15]. However, increases in O_3_ concentrations, particularly during the first stage, increased mortality in populous eastern China [16].

The health benefits of air pollution control should be maximized, and the environmental inequalities minimized as well. Although interpretation on environmental inequality can be complex (for more details, please see the discussion section), in this study, we utilized the terminology to describe the phenomenon that a large fraction of exposed population, disease burden, or another additive measure of environmental impact is disproportionally attributable to a small identifiable subgroup. In high-income countries, actions targeting toward environmental equality, such as the Justice40 Initiative in the United States (US), have been taken to identify disadvantaged communities, and to prioritize them for public policies on air pollution control and climate mitigation [17]. There is considerable inequality embedded in exposure to, and the health effects of, ambient air pollution, particularly in low- and middle-income countries (LMICs) [18]. For instance, in China, exposure to nitrogen dioxide (NO_2_) and PM_2.5_ tends to scale with increasing socioeconomic status [19]. However, few studies have evaluated this trend. In our previous study, we assessed attributable deaths linked to long-term exposure to NO_2_, and the geographic inequality therein. The distribution of NO_2_-related deaths was disproportional, with the top 20% high-risk individuals contributing 85.7% of attributable deaths [20]. In addition to the attributable burden of air pollution exposure, policymakers and the public are also interested in the relevant inequality. For instance, PM_2.5_ exposure in the US exhibits inequalities among races and income levels. In addition, from 2000 to 2016, the fraction of the population exposed to PM_2.5_ >8 μg/m^3^ decreased from 89% to 41% as the degree of inequality between racial groups gradually increased. Therefore, the health benefit of a reduction in air pollution may not be distributed equally [21], and warrants a study to quantitively examine whether a strategy of environmental management increases or decreases such inequalities, particularly among the LMICs, such as China.

Environmental inequality can have several causes, and intervening in some of those could be prohibitively costly. For instance, individuals living close to desert areas are more frequently affected by exposure to dust particles; migration is the ultimate solution to this but may be unaffordable. Therefore, investigations of the distributions of the absolute health effects of air pollution exposure [20] may overestimate environmental health inequality. It would be useful to assess how temporal changes in air pollution-related disease burden (also known as relative disease burden) are differentially geographically distributed. Additionally, to establish air pollution-control targets at the population level and to optimize their health benefits [22], stakeholders are interested in the geographic inequality (also known as spatial inequality or spatial disparity) caused by regional differences in, for instance, population characteristics. However, no study has assessed the geographic inequalities of the health benefits caused by CAAs.

In this study, we developed a health metric (gain of life expectancy (GLE)) to monitor the health benefits associated with CAAs based on classical risk assessments, and evaluated the geographic inequality embedded in the metric using Lorenz curves and the Gini index (GI). The Lorenz curve visualizes cumulative distribution of the health benefits against the corresponding population distribution, and GI is a summary statistic of the curve. The risk assessments focused on the long-term exposures to PM_2.5_ and O_3_, which had been used as additive risk factors in previous studies [23–25]. We utilized the metric of attributable deaths or years of life lost (YLL) to measure the sum of health impact from air pollution exposure among different subgroups, distinguished by sex, age, residence, and spatiotemporal coordinate. YLL was further transformed into loss of life expectancy (LLE) to be indicative for the health impact per capita. We investigated the magnitude and inequalities in health benefits during the two stages of the CAAs, from 2013 to 2020.

## RESULTS

### PM_2.5_ and O_3_ exposure

**Figure 1.**
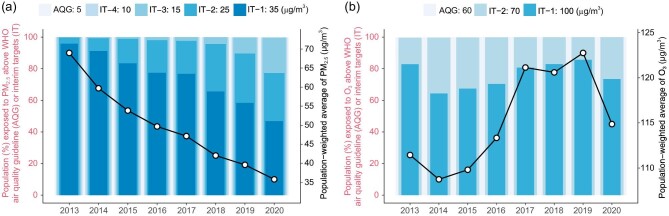
PM_2.5_ (a) and O_3_ (b) exposures in China from 2013 to 2020. The black lines and circles (referring to right *y*-axis) show nationwide population-weighted average exposure, and the colored bars (referring to left *y*-axis) show percentages of population exposed to pollution above levels recommended by the World Health Organization (WHO). We also present the least-square trends in exposure by grid in Supplementary Fig. S1.

The population-weighted concentration of PM_2.5_ decreased from 68.98 μg/m^3^ to 47.13 μg/m^3^ in the first stage of the CAAs and to 35.77 μg/m^3^ after the second stage (Fig. 1). The proportion of adults exposed to a polluted level of PM_2.5_ (greater than the national ambient air quality standard, i.e. 35 μg/m^3^, equal to the first WHO interim target) decreased from 95.86% to 76.77% and 46.83% after the first and second stages, respectively. The gridded map of trends from 2013 to 2020 indicated geographic heterogeneity in the effect of CAAs on PM_2.5_ exposure (Supplementary Fig. S1).

The values for O_3_ increased from 111.4 μg/m^3^ to 121.1 μg/m^3^ in the first stage and decreased slightly to 114.9 μg/m^3^ after the second stage. The proportion of adults exposed to a polluted level of O_3_ showed a similar temporal trend. A gridded map of trends showed that hotspots of O_3_ growth spanned north and northwest China (Supplementary Fig. S1), suggesting considerable geographic inequality in the effect of CAAs on O_3_ exposure.

### Attributable burden of mortality

**Figure 2.**
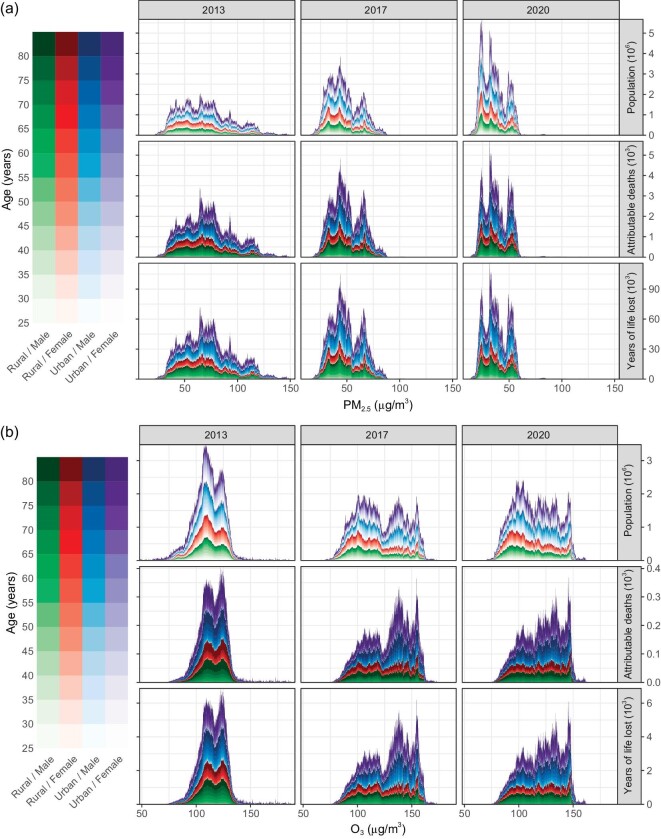
Distributions of exposure to and the health effects of PM_2.5_ (a) and O_3_ (b) by sex, age, and area of residence among Chinese adults in 2013, 2017, and 2020. For more distributions standardized by sex, age, and residence, please see Supplementary Fig. S2.

Figure 2 shows the population distribution by exposure level or health impact of air pollutants in 2013, 2017, and 2020. In 2013, 1.31 (95% confidence interval [CI]: 1.26–1.39) million attributable deaths and 28.87 (95% CI: 27.88–30.71) million YLLs were associated to PM_2.5_ exposure. These values decreased to 1.23 (95% CI: 1.14–1.26) and 26.97 (95% CI: 24.90–27.60) in 2017 and to 1.06 (95% CI: 1.00–1.12) and 22.62 (95% CI: 21.41–23.92) in 2020, respectively. Compared to PM_2.5_, O_3_ exposure made a smaller contribution to the burden of mortality. In 2013, 0.102 (95% CI: 0.100–0.103) million attributable deaths and 1.76 (95% CI: 1.72–1.77) million YLLs were associated to O_3_ exposure. These values increased to 0.124 (95% CI: 0.121–0.125) and 2.10 (95% CI: 2.05–2.17) in 2017 and decreased to 0.116 (95% CI: 0.113–0.116) and 1.93 (95% CI: 1.88–1.94) in 2020, respectively. For PM_2.5_ and O_3_, the burden on males was heavier than on females, that on the elderly was heavier than on the young, and that on urban residents was heavier than on rural residents.

### Health benefits

The burden of mortality was dominated by PM_2.5_ exposure, which had LLEs of 1.86 (95% CI: 1.80–1.98), 1.69 (95% CI: 1.56–1.72), and 1.38 (95% CI: 1.30–1.46) in 2013, 2017, and 2020, respectively. Based on only the PM_2.5_-associated disease burden, the first and second stages of CAAs contributed to GLEs of 2.11 and 3.68 months, respectively. Combining the health effects of PM_2.5_ and O_3_ exposure, the GLE was estimated to be 1.87 months for the first stage and 3.94 months for the second stage of CAAs.

**Figure 3.**
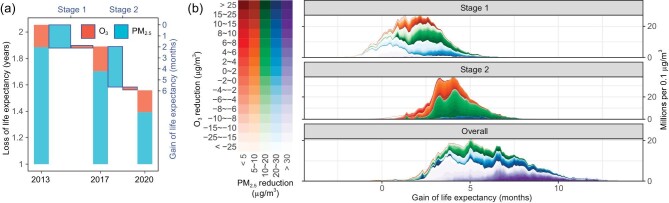
Gain of life expectancy (GLE) caused by CAAs, 2013 to 2020. (a) Temporal trend in GLE, determined by changes in loss of life expectancy attributable to PM_2.5_ and O_3_ exposure. (b) Distributions of GLE by level of air-quality improvement, codetermined by PM_2.5_ and O_3_ reductions. The detailed geographic distributions of GLE are documented in Supplementary Fig. S3.

Figures 3 and S3 show the distributions of GLE associated with CAAs. During the first stage, GLE was distributed unevenly, being low in the north and high in the south. Although the PM_2.5_ concentrations in the North China Plain and Fenwei Plain (NCP-FP) decreased markedly during the first stage, the GLE of joint exposure to PM_2.5_ and O_3_ was low for two reasons. First, the increment in O_3_ exposure offset the health benefits caused by PM_2.5_ reduction. Second, due to the sublinear curvature of the relationship between PM_2.5_ and mortality, the marginal effect on health of the same reduction from baseline of high-concentration exposure was smaller than that for low-concentration exposure. By contrast, during the second stage, GLE was evenly distributed geographically. Throughout CAAs, GLE was high in the YRD and Sichuan Basin. Similarly, because of the synergic control of PM_2.5_ and O_3_ and the improved baseline air quality, the GLE was higher in the second than the first stage, although the reduction in PM_2.5_ concentration in the second stage (11.36 μg/m^3^) was smaller than in the first stage (21.85 μg/m^3^).

**Figure 4.**
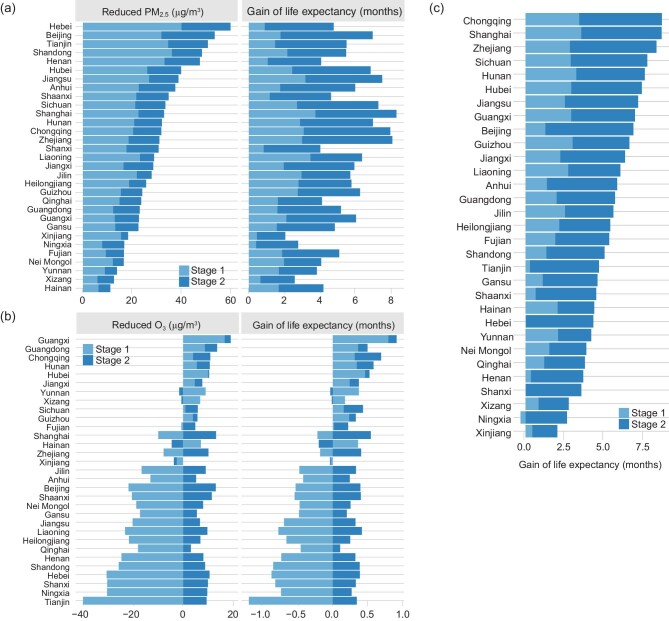
Gain of life expectancy during CAAs by province, 2013 to 2020. (a) and (b) Improvements in air quality and health for PM_2.5_ and O_3_, respectively. (c) Total gain of life expectancy attributable to PM_2.5_ and O_3_ reductions in combination.

Analyses by province yielded similar results (Fig. 4). The provincial average GLEs did not reflect changes in exposure to PM_2.5_. For O_3_, which was linked to mortality by a uniform function for adults of all ages, the provincial average GLEs were proportional to the corresponding changes in exposure. The GLE during CAAs was codetermined by changes in air pollutants and demographic characteristics, as well as their nonlinear interactions. The average baseline mortality rate, standardized by the 2013 demographic structure, was reduced from 7.6 per 1000 adults in 2013 to 6.9 in 2020, meaning that the population became less vulnerable due to improvements in many aspects such as healthcare and medical accessibility. The decreased vulnerability would shrink the health benefits of CAAs. In contrast, without standardization by demographic structure, the raw baseline mortality rate was increased to 7.8 per 1000 adults in 2020. The different trend in mortality rate during 2013–2020 after standardization showed the changes in demographic structure, particularly population aging (Supplementary Fig. S2). Briefly speaking, the rapidly aging population in China increased the sensitivity to air pollution, and thus would amplify the health benefits of CAAs.

### Geographic inequality embedded in health benefits

**Figure 5.**
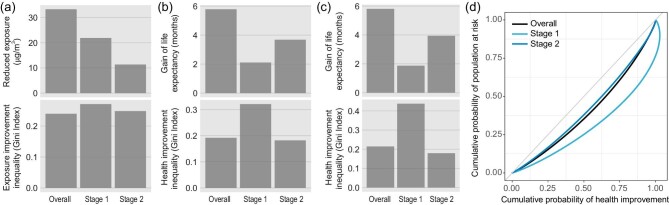
Magnitudes of geographic inequality embedded in the health benefits at the two stages of CAAs. (a) Improvement in PM_2.5_ exposure (top panel) and Gini indexes for their geographic inequalities (bottom panel). (b) PM_2.5_-attributable health benefits (top) and its inequality (bottom). (c) Health benefits (top) and its inequality (bottom) attributable to PM_2.5_ and O_3_ in combination. (d) Lorenz curves underlying the Gini indexes in (c).

Use of different air pollution-control targets and their interactions with spatially varying demographic characteristics resulted in a complex geographic pattern in CAA-mediated health benefits (Fig. 2). During the first stage, half of the health benefits was evident in 25% of the population (GI 0.44). This increased to 37.5% during the second stage (GI 0.18). The degree of inequality embedded in the reduced PM_2.5_ concentration was stable (Fig. 5). In other words, the decreased inequality in health benefits could be partially attributable to control of O_3_ pollution. Furthermore, as shown in Fig. 2, the burden of diseases was disproportionally distributed between different age groups. As expected, a large fraction of attributable deaths was highly clustered among the elderly, who are recognized as being susceptible to cardiorespiratory diseases. Given the trend of population aging in China (Supplementary Fig. S2), the increased sensitivity to the beneficial effects of air pollution reduction can also partially explain the decreased geographic inequality.

## DISCUSSION

To the best of our knowledge, this is the first assessment of the effect of CAAs on health benefits and health equality. Compared to the first stage of the CAAs, the second stage had a smaller reduction in PM_2.5_ exposure but a greater benefit in health, which was distributed more equally. Therefore, CAAs not only reduced attributable deaths but also improved relevant health equality. Our findings will facilitate the optimization of air pollution-control policies to better protect public health.

### Sensitivity to the health effects of air pollution

The health benefits of CAAs were codetermined by air pollution control and human sensitivity. In this study, human sensitivity was captured by baseline mortality, age structure, and baseline exposure level. Subgroups with higher baseline mortality, advanced age, and lower PM_2.5_ exposure were more sensitive to air quality changes. High sensitivity could enhance the health benefits caused by air pollution reduction, explaining the greater health benefits in the second stage of the CAAs. Therefore, Chinese adults are increasingly sensitive to air pollution exposure.

Other factors contribute to sensitivity to the health effects of air pollution. First, chemical profiles modify the toxicity of PM_2.5_, thereby modulating the health effects. For instance, desert dust, biofuel burning, and open fires were the three largest contributors to PM_2.5_-related deaths in children under 5 years of age [26]. Similar results have been reported for birthweight reductions attributable to PM_2.5_ [27]. Second, sociodemographic factors can affect susceptibility or vulnerability to the adverse effects of PM_2.5_ and O_3_. Bell *et al*. systematically reviewed epidemiological evidence on susceptibility to morbidity or mortality related to short-term O_3_ exposure. Elderly individuals were significantly more susceptible to O_3_ exposure (excess risk 1.27%, 95% CI: 0.76–1.78) than young people (0.60%, 95% CI: 0.40–0.80). Individuals of low socioeconomic status (such as unemployed/low occupational status, ethnic/racial minorities, low educational level, and poverty) are potentially susceptible to O_3_ exposure [28]. Findings on susceptibility to the short-term health effects of PM_2.5_ are similar [29]. Third, behavioral factors can also modify the association between ambient exposure and a health outcome by affecting personal exposure patterns such as respiration rate. For instance, active commuting enhances, and physical activity attenuates, the association between PM_2.5_ exposure and cerebrovascular disease. By ignoring such factors, we might have underestimated the magnitude of geographic inequality.

Additionally, we might have underestimated geographic inequality by ignoring adverse effects on newborns and children, who are sensitive to air pollution. Gestational PM_2.5_ or O_3_ exposure increases the risk for pregnancy loss [30–32], but there is little evidence on this risk in China. According to a previous study, each 10 μg/m^3^ increment of PM_2.5_ exposure during pregnancy increases the risk for stillbirth by 10% (95% CI: 7–13) [31]. In China, PM_2.5_ and O_3_ adversely affect birth outcomes, leading to preterm birth and low birthweight outcomes [33–36]. For example, in a study, each 10 μg/m^3^ increment of gestational PM_2.5_ exposure was associated with a 26.2% (95% CI: 8.7–46.5) increased risk for preterm birth [33]. In another study, each 10 μg/m^3^ increment of gestational O_3_ exposure increased the risk for term low birthweight by 3% (95% CI: 0.1–5.2) [36]. Moreover, acute exposure to PM_2.5_ could increase the risk of mortality in children under 5 years of age in China, with a greater effect in neonates [37]. However, the lack of gridded data on the baseline prevalence of stillbirth, low-birthweight livebirth, or preterm birth enabled us to estimate the disease burden attributable to PM_2.5_ and O_3_ among adults only.

### Policy implications

The high degree of inequality in the first stage of the CAAs may be explained by the slight improvement in health across some regions, such as NCP-FP. In the second stage, one focus was controlling O_3_ pollution by synergically reducing emissions of VOCs and nitrogen oxides (NO_x_), which enhanced the health benefits. In contrast, the lack of VOC control, particularly in urban areas, the reduction in NOx concentration [38] contributed to an increased O_3_ exposure during the first stage, due to VOC-limited regimes [39]. It is worth mentioning that some studies have proven a positive interaction between the health effect of PM_2.5_ and that of O_3_ [40]. The PM_2.5_-O_3_ interaction is also known as the synergic health effect. Given that, the burdens of diseases separately estimated for PM_2.5_ and O_3_ are not additive, and the current method might even underestimate the health benefits brought by the synergic emission control for PM_2.5_ and O_3_. Furthermore, it is worth mentioning that the synergic strategies could change source sectors and chemical compositions of the PM_2.5_ mixture. For instance, the fraction of VOC in PM_2.5_ mixture and VOC-related sources (e.g. diesel vehicular exhaust) will be reduced, in order to synergically control for O_3_. However, the current risk assessment approach ignored inequal toxicities between different PM_2.5_ components, and thus couldn’t evaluate the health impacts from the synergic emission controls, accurately.

Additionally, with the in-depth reduction in PM_2.5_ pollution, residents in heavily polluted areas, such as NCP-FP, became sensitive to the resulting beneficial effects. Furthermore, the second stage focused on three key regions likely contributing to the reduced geographic inequality (NCP, FP, and YRD). In terms of maximizing the health benefits or minimizing the inequality therein, the second stage of CAAs outperformed the first stage. Therefore, optimized strategies including the synergic control of PM_2.5_ and O_3_, and in-depth reduction in air pollution, should be considered for future air quality management.

Although the CAAs in China can promote equality, warranted are further additional investigations and policies specifically targeted to minimizing environmental injustice. In high-income countries, such strategies have been put into action. For instance, the recent Integrated Science Assessment for particulate matter and its supplement, the scientific reports to support the proposal to strengthen the annual PM_2.5_ standard in the US from 12 μg/m^3^ to 9–10 μg/m^3^, had identified disadvantaged subpopulations by socioeconomic status and race (https://www.federalregister.gov/d/2020-01223 and https://www.federalregister.gov/d/2022-07938). The US Environmental Protection Agency also proposed to modify the PM_2.5_ monitoring network design criteria to include an environmental justice factor. The Justice40 Initiative developed the Climate and Economic Justice Screening Tool (CEJST) to identify disadvantaged communities and to allocate future resources for environmental improvement. Although studies have found CEJST could not effectively reduce inequalities embedded in air pollution exposure [17], studies of quantitative inequality assessment, like ours, will contribute to develop an optimized tool, and to design justice policies.

### Complexities underlying environmental inequality

Improving environmental equality is an aim of environmental governance. There is growing interest in the distribution of the effects of environmental policies [41,42]. However, the concepts of equality are often interpreted and operationalized differently. As a result, multiple dimensions or indicators of inequality are needed to evaluate air pollution-control policies [43].

To achieve equal distribution of clean air, it has been assumed that all individuals should be exposed to an equal level of air pollution. Therefore, exposure levels by subpopulations, such as multiple ethnic groups, need to be examined. For instance, a previous study combined US demographic and PM_2.5_ data from 2004 to 2016, and examined whether minority (Black, Asian, and Hispanic or Latino) areas had higher levels of PM_2.5_ [21]. Second, to achieve equality in terms of individual health, it has been assumed that the disease burden attributable to air pollution should be equally borne by all individuals. Therefore, health inequalities, such as the effect of air pollution on mortality and hospitalization, need to be examined. For example, Fann *et al*. evaluated the effects of air pollution-control strategies on the inequalities of mortality and the risk for hospitalization due to asthma [44]. Xue *et al*. studied the inequalities in the distributions of attributable deaths and YLLs caused by NO_2_ exposure in China from 2013 to 2020 [20]. Third, equality can also be interpreted to mean that the exposure level or health effects of air pollution should be proportional to socioeconomic status. This is based on the criterion of equality in promoting the welfare of socially disadvantaged or vulnerable groups. For example, Tomar *et al*. examined whether lower socioeconomic status groups experience more premature deaths due to PM_2.5_ exposure in India [45]. Finally, inequality has also been utilized to describe that different source sectors disproportionally contributed to the burden of air pollution, due to the unequal toxicities for different chemical components [46]. However, to reveal such inequality, source- or component-specific air pollution concentrations and customized joint-exposure-response functions [47] are warranted, which is beyond the capability of commonly-utilized risk assessment approaches.

Policies with a focus on the abovementioned inequalities are critical for promoting environmental equality and justice. Although the CAAs improved air quality and alleviated the associated health burdens, the distribution of health benefits had not been examined, before this study. We evaluated the geographic inequality of the CAAs, which assumed that the health benefits of an air pollution-control policy are equally shared by all individuals. Our findings provide insights into the equitable geographic distribution of clean air and health benefits. However, we did not consider detailed demographic and sociological characteristics or assess health benefits in vulnerable groups. Therefore, further investigation is needed.

## CONCLUSION

The health effects of the second stage of CAAs were greater than the first stage, in terms of both increased efficacy and decreased inequality. Our findings suggest that continuously improving air quality throughout China will benefit public health.

## METHODS

### Environmental and population data

PM_2.5_ and O_3_ concentration (1 × 1 km resolution) data were taken from previous studies [48,49], which combined multiple datasets using machine-learning algorithms (random forest, extremely randomized trees, and extreme gradient boosting). The inputs were satellite atmospheric measurements, meteorological fields, chemical-transport model simulations, and other geographical variables. The models for predicting daily levels of PM_2.5_ and O_3_ in China were evaluated by cross-validation based on monitoring data, and their overall performances were good (*R*^2^ 0.92 and 0.89 for long-term concentrations of PM_2.5_ and O_3_, respectively) [48,49].

We analyzed the effects of PM_2.5_ and O_3_ on health, considering factors such as sex, age, and place of residence. Therefore, population maps based on sex and age were combined with urbanization maps. We used WorldPop (https://www.worldpop.org/) data to obtain information on sex and age, which were combined and aggregated into a regular 1 × 1 km grid over China. Population data were estimated by combining spatial population datasets with national age and sex data, as described previously [50,51]. An urbanization map of China was obtained from satellite images of impervious surfaces using a product developed by Gong *et al.* [52]. We combined those maps to develop a series of gridded maps for each subpopulation based on sex, age, and urban/rural residence. For details of data processing, see a previous study [20].

### Assessment of mortality burden

We applied the method developed by the 2019 GBD Study to assess the mortality burden attributable to long-term ambient air pollution exposure among all Chinese adults (>25 years old) from 2013 to 2020. The risk assessments for long-term PM_2.5_ and O_3_ exposures were performed within a 1 × 1 km grid by subpopulations of sex, age, and residence (urban or rural), and were parametrized using the following equations:


(1) \begin{eqnarray*}
{{\mathrm{D}}}_{s,t,k} &=& {\mathrm{AF}}({{\mathrm{C}}}_{s,t}) \times {{\mathrm{B}}}_{s,k} \times {{\mathrm{P}}}_{s,t,k},\\
{\mathrm{AF}}({{\mathrm{C}}}_{s,t}) &=& 1-1 \div {\mathrm{R}}{{\mathrm{R}}}_{s,t,k},\\
{\mathrm{R}}{{\mathrm{R}}}_{s,t,k} &=& {\mathrm{MR}} \hbox{-} {\rm {BR}}{{\mathrm{T}}}_k({{\mathrm{C}}}_{s,t})\\
&& \div {\mathrm{MR}} \hbox{-} {\rm {BR}}{{\mathrm{T}}}_k\left( {{\mathrm{TMREL}}} \right),
\end{eqnarray*}

where subscripts *s, t*, and *k* denote the indexes for spatial grid, calendar year, and sex-age-residence subpopulations, respectively; D*_s_,_t_,_k_* represents the attributable deaths among the *k^th^* subpopulation in the *s^th^* grid cell during the *t^th^* year; C, B, and P are the three inputs, i.e. annual concentration of PM_2.5_ or O_3_, baseline mortality rate, and population size, respectively; AF is the attributable fraction; RR is the relative risk; MR-BRT is the nonlinear exposure-response function; and TMREL is the theorical minimum risk exposure level. The Meta-regression–Bayesian, Regularized Trimmed (MR-BRT) tool estimates the function that links a risk factor to mortality risk. The MR-BRT and TMREL parameters were obtained from the 2019 GBD Study [23]. The MR-BRT considers six cause-specific deaths attributable to PM_2.5_ exposure—ischemic heart disease, stroke, lung cancer, chronic obstructive pulmonary disease (COPD), lower-respiratory-tract infection, and type-2 diabetes for age-specific adults—and considers COPD-caused deaths for long-term O_3_ exposure. The long-term concentration (C*_s_,_t_*) was set as the 12-month average PM_2.5_, or the peak-season value of O_3_. We referred to the recent air quality guidelines of the World Health Organization to evaluate long-term O_3_ exposure [53]. We obtained the daily 8 h average from the gridded estimates and calculated the monthly averages. Then the peak-season value was calculated as the maximum 6-month moving average, based on the monthly average concentrations. The premature deaths associated with peak-season O_3_ concentrations have been reported [54]. We also obtained gridded estimates of baseline total mortality rates by combining the data in the annual reports of the National Disease Surveillance Program and the gridded maps for *sex-age-residence* subpopulations. The gridded mortality estimates were validated against the county-level values in the census data. We used the mortality causes profile from the GBD Study [23] to obtain disease-specific baseline estimates.

### Assessment of health benefits by gain of life expectancy

We transformed the number of attributable mortalities to loss of life expectancy (LLE), a disease burden metric that is scalable and computationally additive. LLE can be estimated using the following equation:


(2) \begin{eqnarray*}
{\mathrm{LL}}{{\mathrm{E}}}_{s,t} &=& {\mathrm{L}}{{\mathrm{E}}}_0 \times {\mathrm{YL}}{{\mathrm{L}}}_{s,t} \div {{\mathrm{P}}}_{s,t},\\
{{\mathrm{P}}}_{s,t} &=& {\sum }_k{{\mathrm{P}}}_{s,t,k},\\
{\mathrm{YL}}{{\mathrm{L}}}_{s,t} & =& {\sum }_k{{\mathrm{D}}}_{s,t,k} \times {\mathrm{L}}{{\mathrm{E}}}_k,
\end{eqnarray*}

where LE*_k_* denotes the theorical life expectancy at a given age for the *k^th^* subpopulation and YLL*_s_,_t_* represents years of life lost. The LLE at birth can be viewed as the re-scaled average of the YLL given a specific age-structure and has been used in previous works [20,55]. To quantify health benefits, we derived an indicator, gain of life expectancy (GLE):


(3) \begin{eqnarray*}
{\mathrm{GL}}{{\mathrm{E}}}_s &=& {\mathrm{LL}}{{\mathrm{E}}}_{s,t = y1}-{\mathrm{LL}}{{\mathrm{E}}}_{s,t = y2}\\
&=& {\mathrm{L}}{{\mathrm{E}}}_0({\mathrm{YL}}{{\mathrm{L}}}_{s,t = y1} \div {{\mathrm{P}}}_{s,t = y1}\\
&& -{\mathrm{YL}}{{\mathrm{L}}}_{s,t = y2} \div {{\mathrm{P}}}_{s,t = y2})\\
&=& {\mathrm{L}}{{\mathrm{E}}}_0[({{\mathrm{P}}}_{s,t = y1} \div {{\mathrm{P}}}_{s,t = y2}){\mathrm{YL}}{{\mathrm{L}}}_{s,t = y1}\\
&& -\,{\mathrm{YL}}{{\mathrm{L}}}_{s,t = y2} \equiv {\mathrm{YL}}{{\mathrm{G}}}_s] \div {P}_{s,t = y2}\\
&=& {\mathrm{L}}{{\mathrm{E}}}_0 \times {\mathrm{YL}}{{\mathrm{G}}}_s \div {P}_{s,t = y2},
\end{eqnarray*}

where *y*1 and *y*2 are the temporal indexes for the beginning and ending years of air pollution control, respectively (e.g. for the first stage of CAAs, *y*1 = 2013 and *y*2 = 2017), and YLG denotes years of life gain, which measures the reduction in YLL with adjustment for population size.

### Assessment of inequality

We viewed the CAA-associated GLE as a type of *health income*, and used the classical econometric method to evaluate the relevant inequality. We created a Lorenz curve to assess the geographic inequality embedded in the health benefits (GLE*_s_*) caused by a reduction in PM_2.5_ exposure, O_3_ exposure, or their combination, during a given stage of air pollution control. The Lorenz curve, a graphical representation of income distribution that plots the cumulative share of total income held by a given percentage of the population, is expected to be a diagonal line, indicating absolute equality. Correspondingly, to generate health Lorenz curves, income was replaced by the health benefits metric (i.e. GLE). The coordinates (*x_i_, y_i_*) of points on health Lorenz curves can be expressed as:


(4) \begin{eqnarray*}
{x}_i &=& {\sum }_{s \in \Omega (i)}({\mathrm{GL}}{{\mathrm{E}}}_s \times {P}_{s,t = y2})\\
&& \div {\sum }_s({\mathrm{GL}}{{\mathrm{E}}}_s \times {P}_{s,t = y2})\\
&=& {\sum }_{s \in \Omega (i)}{\mathrm{YL}}{{\mathrm{G}}}_s \div {\sum }_s{\mathrm{YL}}{{\mathrm{G}}}_s,\\
{y}_i &=& {\sum }_{s \in \Omega (i)}{{\mathrm{P}}}_{s,t = y2} \div {\sum }_s{P}_{s,t = y2}\\
&&{\Omega {{(i)}}:\forall s,\quad{\mathrm{GL}}{{\mathrm{E}}}_s < {c}_i}
\end{eqnarray*}

where Ω(*i*) denotes the subset of the population with a GLE below a given constant level (*c_i_*). We calculated the GI to measure the deviation from the diagonal line, where a greater value indicates a more disproportionate distribution of health benefits.

Analyses were performed in R (version 4.2.2). We used the Monte Carlo approach to evaluate the uncertainties embedded in the mortality-burden metrics, and calculated the empirical CIs. Due to computational complexity, we were unable to calculate uncertainties for the Lorenz curves and GIs.

## Supplementary Material

nwad263_Supplemental_File
