# Supplementary material for: Health benefits from the rapid reduction in ambient exposure to air pollutants after China's clean air actions: progress in efficacy and geographic equality
Source: Natl Sci Rev. 2023 Oct 9;11(2):nwad263. doi: 10.1093/nsr/nwad263 (PMC10776362; doi:10.1093/nsr/nwad263)
Supplement: nwad263_Supplemental_File [file nwad263_supplemental_file.docx]

***Supplemental materials of***

**Health Benefits from the Rapid Reduction in Ambient Exposure to Air Pollutants after China’s Clean Air Actions: Progress in Efficacy and Geographic Equality**

Xue Tao et al.

(a) Least-square trend in PM_2.5_


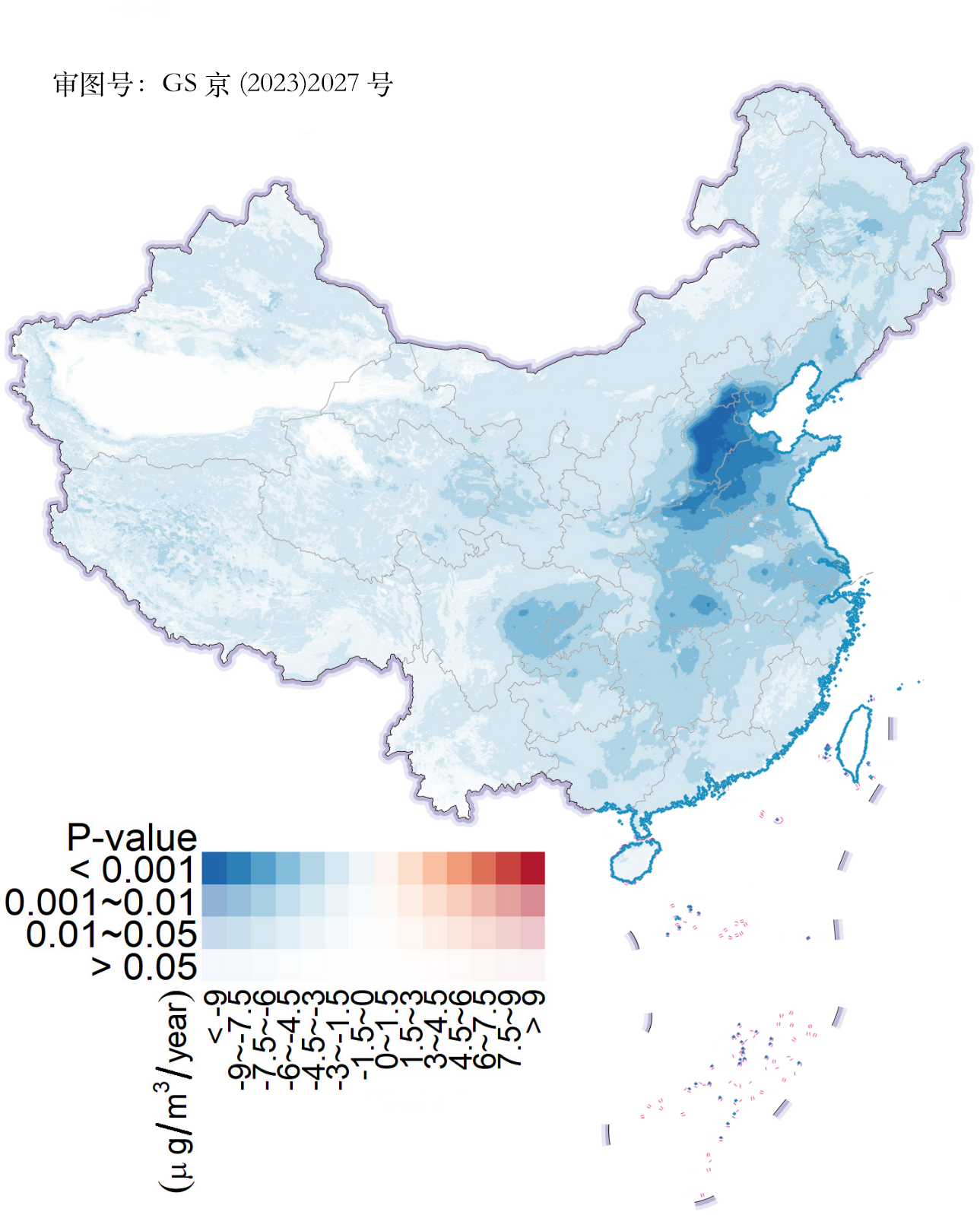


(b) Least-square trend in O_3_
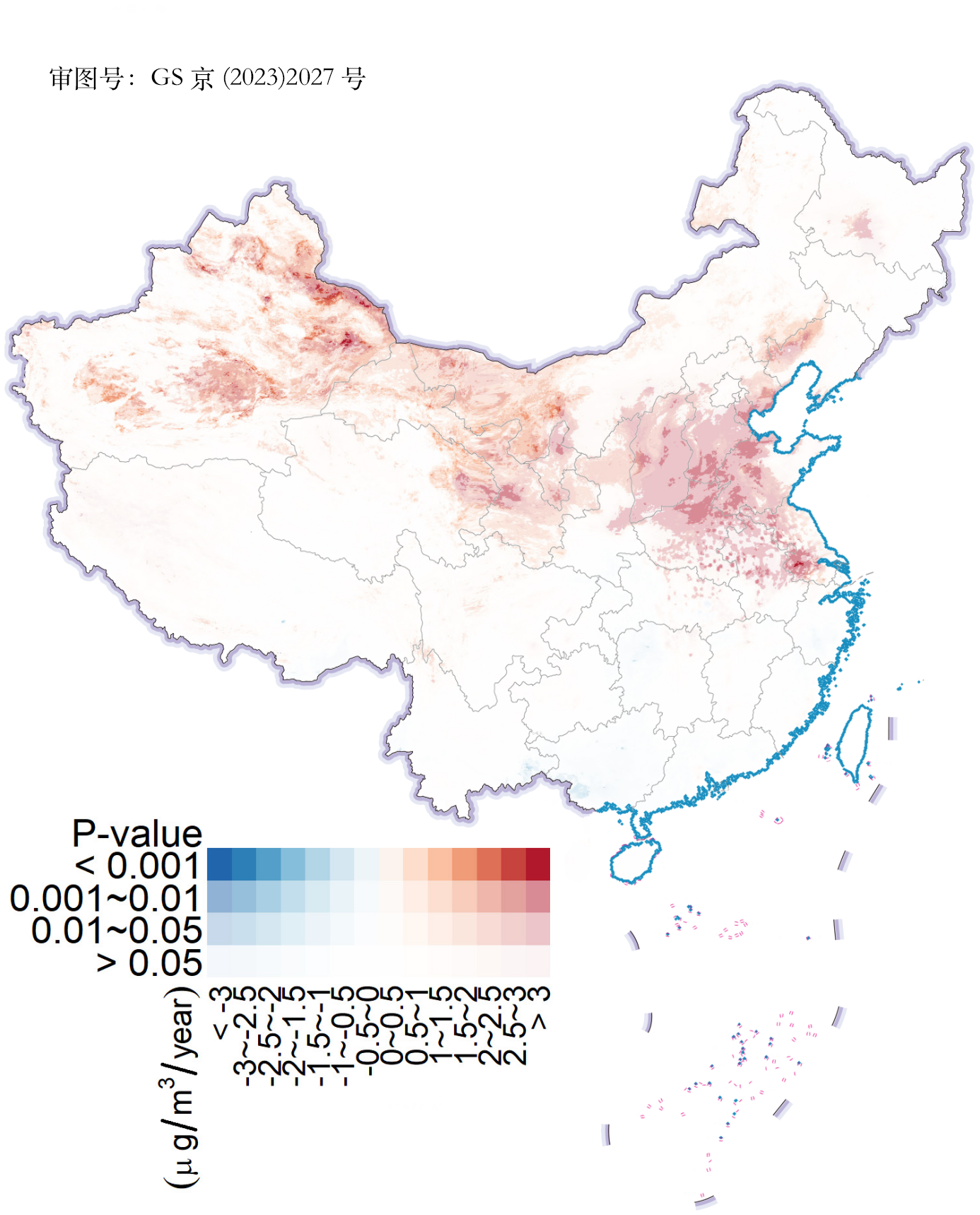


Figure S1 Least-square trends in exposures to PM_2.5_ (a) and O_3_ (b) by grid. Background color represents the point estimate of the least-square trend, and transparency shows the level of statistical significance (P-value).

(a)

(b)

Figure S2 Distributions of inputs (namely, exposure, population, and baseline mortality) and outputs (namely, premature deaths, years of life lost, and loss of the life expectancy) of the diseases burden assessment for PM_2.5_ (panel a) or O_3_ (panel b) in 2013 (solid lines) and 2020 (dashed lines), standardized by sex, age, and residence.

(a) Overall


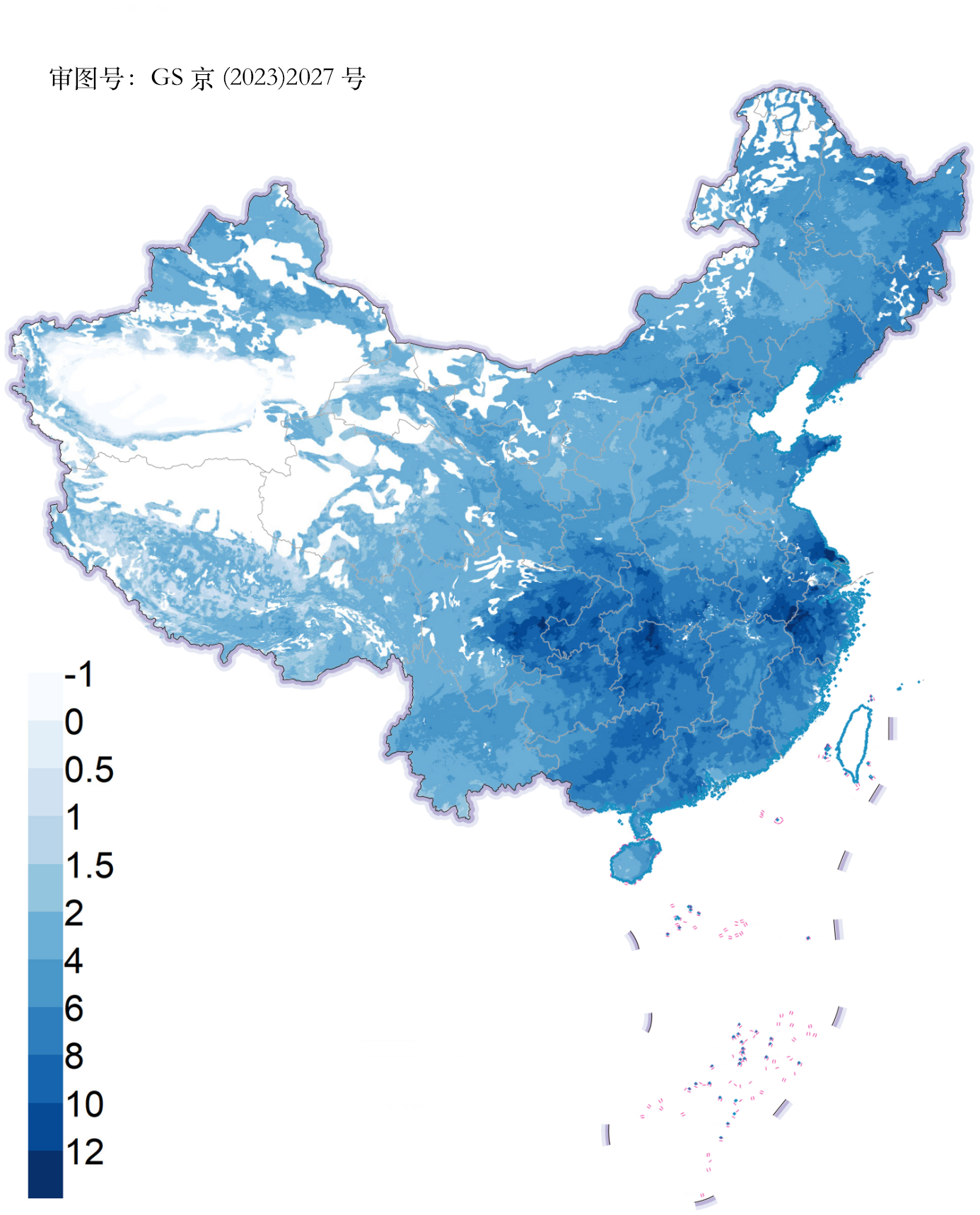


(b) Stage 1


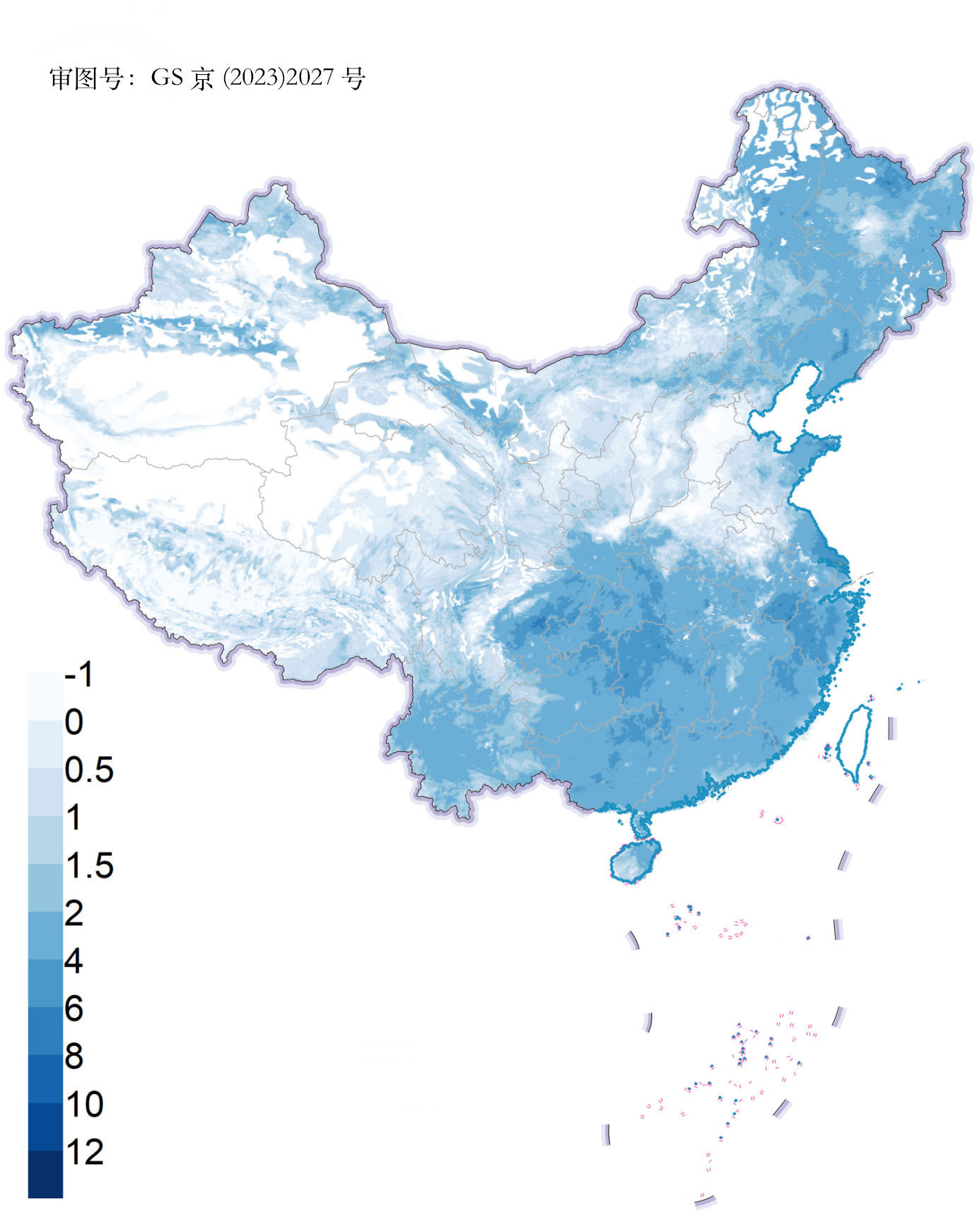


(c) Stage 2


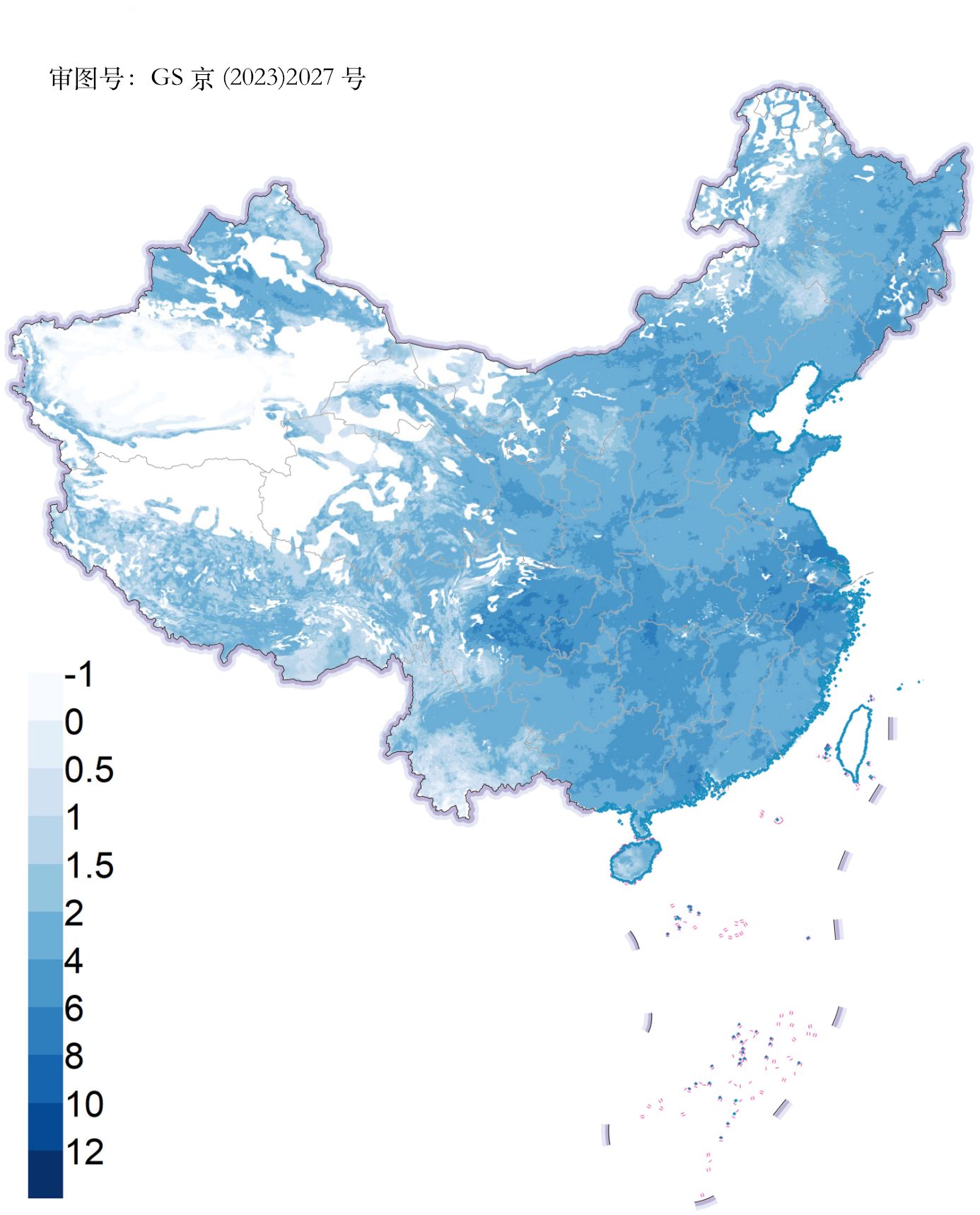


Figure S3 Geographic distributions for the gain of life expectancy (GLE) caused by CCAs, 2013 to 2020.
